# *δ*-Aminolevulinic acid transport in murine mammary adenocarcinoma cells is mediated by beta transporters

**DOI:** 10.1038/sj.bjc.6600481

**Published:** 2002-08-12

**Authors:** M Bermúdez Moretti, S Correa García, C Perotti, A Batlle, A Casas

**Affiliations:** Centro de Investigaciones sobre Porfirinas y Porfirias (CIPYP), FCEN, University of Buenos Aires, CONICET. Ciudad Universitaria, Pabellón II, 2° Piso, 1428, Buenos Aires, Argentina

**Keywords:** δ-aminolevulinic acid, membrane transport, photodynamic therapy, BETA transporters

## Abstract

δ-aminolevulinic acid, the precursor of porphyrin biosynthesis has been used to induce the endogenous synthesis of the photosensitiser protoporphyrin IX for photodynamic therapy in the treatment of various tumours. The aim of this work was to characterise the δ-aminolevulinic acid transport system in the murine mammary adenocarcinoma cell line LM3 using ^14^C-δ-aminolevulinic acid, to finally improve δ-aminolevulinic acid incorporation in mammalian cells. Our results showed that δ-aminolevulinic acid is incorporated into these cells by two different mechanisms, passive diffusion which is important at the beginning of the incubation, and active transport. Specificity assays suggested that the transporter involved in δ-aminolevulinic acid incorporation is a BETA transporter, probably GAT-2.

*British Journal of Cancer* (2002) **87**, 471–474. doi:10.1038/sj.bjc.6600481 www.bjcancer.com

© 2002 Cancer Research UK

## 

Photodynamic therapy (PDT) is a non-thermal technique for inducing tissue damage with light following administration of a light-activated photosensitising drug which can be selectively retained in malignant or diseased lesions relative to normal adjacent tissue (Dougherty *et al*, 1978; Hsi *et al*, 1999). In addition, the fluorescence of photosensitising chromophores has been exploited for the visualisation and diagnosis of early stage superficial cancers (Kriegmair *et al*, 1996).

The exogenous administration of δ-aminolevulinic acid (ALA) is a relatively new approach in PDT (Kennedy *et al*, 1990; Fukuda *et al*, 1993). ALA is a natural precursor of protoporphyrin IX (PPIX) which is an intermediate in the haem biosynthetic pathway. Since conversion of PPIX to haem is a rate-limiting step, the exogenous administration of ALA can induce significant intracellular levels of PPIX, which is an effective photosensitiser.

The success of so called ALA-based PDT will depend on the efficient incorporation of ALA into cells as well as an efficient convertion into porphyrins.

ALA-induced PPIX accumulation has been shown to be preferentially greater in certain tumoural cells (Navone *et al*, 1988) primarily due to the reduced activity of ferrochelatase, the enzyme responsible for the conversion of PPIX into haeme (van Hillesberg *et al*, 1992) and a relative enhancement of porphobilinogen deaminase activity (Navone *et al*, 1991), which constitutes the biological rationale for the clinical use of ALA-PDT.

The knowledge of the mechanism of entrance of ALA into the cells will provide new tools to improve ALA-PDT.

Recently, several reports about ALA uptake systems have appeared. Thus, some authors postulated that ALA is taken up through the di- and tri-peptide transporters PEPT1 and PEPT2 (Döring *et al*, 1998; Novotny *et al*, 2000; Whitaker *et al*, 2000). Other authors suggested that BETA transporters are involved in ALA transport (Rud *et al*, 2000). The BETA transporter family comprises GAT-1 to GAT-3, BGT-1 and TAUT transport systems (Palacín *et al*, 1998).

The aim of this work was to characterise ALA transport in a murine mammary adenocarcinoma cell line.

## MATERIALS AND METHODS

### Cell line and cell culture

Cell line LM3 (Werbajh *et al*, 1998) derived from the murine mammary adenocarcinoma M3 was cultured in minimum essential Eagle's medium, supplemented with 2 mM L-glutamine, 40 μg gentamycin ml^−1^ and 5% foetal bovine serum, and incubated at 37°C in an atmosphere containing 5% CO_2_. 3.5×10^4^ cells well^−1^ were seeded into 24-well plates and medium was renewed 24 h before the experiment.

### Chemicals

[4-^14^C]ALA hydrochloride and [^14^C(U)]γ-aminobutyric acid (GABA) were from New England Nuclear. ALA, GABA, amino acids, ALA methyl ester, levulinic acid, captopril, succinyl acetone and metabolic inhibitors were obtained from Sigma Chemical Co., St Louis, USA. (R,S)-nipecotic acid was from Aldrich Chemical Company Inc., Milwaukee, USA. Other chemicals were of analytical grade.

### ALA and GABA preparation

Unlabelled ALA or GABA were dissolved in phosphate-buffered saline (PBS) and pH was adjusted to 7.4 with NaOH. [^14^C]ALA and [^14^C]GABA were added so that the final solution contained 0.0222 MBq ml^−1^ and 0.0111 MBq ml^−1^, respectively.

### Uptake measurements

Uptake measurements were performed 72 h after seeding, when cells were nearly confluent. Cells were washed twice with 0.5 ml PBS-0.1% glucose preheated at 37°C and incubated with 0.3 ml radiolabelled ALA or GABA prepared in PBS-0.1% glucose at 37°C. At the indicated times, the reaction was stopped washing cells four times with 0.5 ml ice-cold PBS containing either 1 mM ALA or 1 mM GABA. Then cells were disrupted in 0.1 mM NaOH and transferred to vials containing scintillation fluid (OptiPhase-Hisafe 3, Perkin Elmer, England). Radioactive content of the samples was determined.

### Porphyrin synthesis

Porphyrins accumulated within the cells were extracted twice with 5% HCl, leaving the cells standing for 30 min in the presence of the acid at 37°C. These conditions proved to be the optimal for total PPIX extraction. The excitation and emission wavelengths of light used producing the highest fluorescence were 406 nm and 604 nm, respectively. PPIX (Porphyrin Products, Logan, Utah, USA) was used as a reference standard.

### Cell number

The number of cells seeded per well and the cell number employed for the calculations were determined by counting viable cells with the Trypan blue exclusion method.

### Viability assay

All compounds employed were previously tested for cell toxicity by means of the MTT assay (Mosmann, 1983). Following appropriate treatments, MTT (3-[4,5-dimethylthiazol-2-yl]-2,5-diphenyltetrazoliumbromide) solution was added to each well in a concentration of 0.5 mg ml^−1^, and plates were incubated at 37°C for 1 h. The resulting formazan crystals were dissolved by the addition of dimethyl sulphoxide (DMSO) and absorbance was read at 560 nm.

### Statistic analysis

Quadruplicates were run for each point in every experiment and the values presented are the average of three experiments. The deviation of these values from the mean was less than 7.5%.

## RESULTS

**Figure 1:**
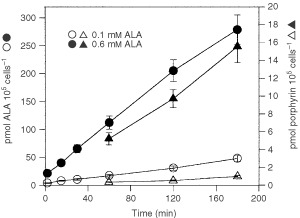
Time course of ALA uptake and porphyrin synthesis. ALA incorporation and porphyrin synthesis were measured incubating cells with 0.1 mM or 0.6 mM C-ALA.

We measured ALA uptake and porphyrin synthesis in cells incubated with 0.1 mM and 0.6 mM ALA (Figure 1

). Initial ALA uptake rate is significantly higher using 0.6 mM ALA (2.26 pmol 10^5^ cell^−1^ min) than using 0.1 mM ALA (0.35 pmol 10^5^ cell^−1^ min). When using 0.1 mM ALA, less than 15% of incorporated ALA was converted into porphyrins after 60 min of incubation, while 40% of incorporated ALA was metabolised into porphyrins from 0.6 mM ALA.

Because the aim of this work was to characterise the ALA transport system in murine mammary adenocarcinoma cells, we tried to minimise the ALA metabolization process during ALA uptake measurement. For this reason we have used 0.1 mM ^14^C-ALA for all transport experiments.

### Specificity of ALA transport system

**Figure 2:**
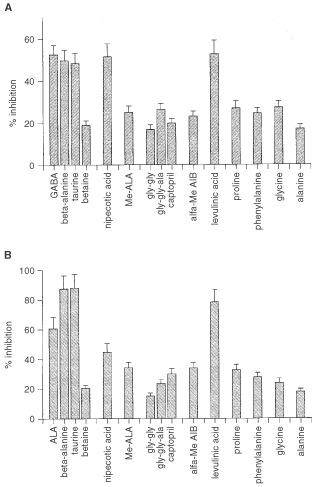
Effect of various compounds on ALA and GABA uptake. Washed cells were preincubated for 15 min with each compound in PBS-0.1% glucose. Then 0.1 mM ^14^C-ALA (**A**) or 0.1 mM ^14^C-GABA (**B**) was added and radioactivity was measured after 30 min. The final concentration of all compounds assayed was 10 mM, except ALA methyl ester and levulinic acid which were 1.25 mM and 3.3 mM, respectively. Values are expressed as percentage of inhibition relative to the control uptake without competitor.

To get an insight into the specificity of the ALA transport system, we measured ALA uptake in the presence of several compounds (Figure 2A

). ALA incorporation is strongly inhibited by GABA and the β-amino acids, β-alanine and taurine, while betaine has no effect. GABA and β-amino acids are substrates of BETA transporters (Palacín *et al*, 1998). Nipecotic acid, an inhibitor of GABA transporters (GAT-1 to GAT-3), diminishes ALA uptake by 50%, whereas the ALA derivative methyl-ALA (Me-ALA) does not. On the other hand, we found a very strong competition of ALA incorporation by its structural analogue, levulinic acid.

The dipeptide glycil-glycine (gly-gly), the tripeptide glycil-glycil-alanine (gly-gly-ala) and captopril, substrates of the transporters PEPT1 and PEPT2, have not any significant effect on ALA uptake.

Because all the substrates of GABA transporters are strong inhibitors of ALA uptake, we analysed GABA uptake in the presence of the same compounds tested for ALA uptake (Figure 2B). As expected, we found that ALA also inhibits GABA uptake. For the rest of the compounds assayed, a pattern of GABA uptake inhibition similar to that obtained for ALA was found (Figure 2A,B), although their effect was higher on GABA than on ALA transport.

### Effect of metabolic inhibitors

To establish the energy dependency of ALA uptake, we measured the uptake of ALA in the presence of different known metabolic inhibitors. When the Na^+^/K^+^ and Na^+^/H^+^ exchange inhibitors, ouabaine and dimethylamiloride, respectively, were used, a moderate decrease in ALA incorporation is observed (Table 1

**Table 1:**
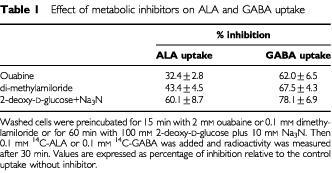
Effect of metabolic inhibitors on ALA and GABA uptake

). When cells were pre-incubated with NaN_3_ (10 mM) and 2-deoxy-D-glucose (100 mM) for 1 h, ALA uptake is reduced by 60%. Under these conditions nearly complete cellular ATP abolishment is achieved (Gederaas *et al*, 2001).

The effect of these inhibitors on GABA uptake is similar to that produced on ALA uptake although greater.

### Dependence of ALA transport on temperature

The above results indicate that ALA incorporation into these cells would be mediated through an active system. However, inhibition by energetic inhibitors never overrides 60% suggesting that a significant uptake would occur by passive diffusion. To test this hypothesis, we compared ALA and GABA transport at 37°C and 4°C. An active transport system would be completely blocked by lowering the temperature to 4°C, whereas involvement of passive transport would imply uptake at low temperatures.

**Figure 3:**
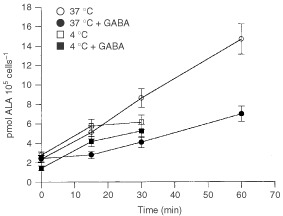
Dependence of ALA uptake on temperature. ALA uptake was measured at 37°C or 4°C in the absence or the presence of GABA. When uptake was determined in the presence of GABA, cells were preincubated 15 min with 10 mM GABA. Then ^14^C-ALA was added and radioactivity was measured.

When ALA uptake is measured at 4°C, a significant incorporation is observed up to 15 min of incubation (Figure 3

) with an initial uptake rate similar to that found in the presence of GABA. Results indicate that the remaining uptake detected at 4°C is not mediated by the active system shared by ALA and GABA.

The time course of ALA uptake at 4°C suggests that during the first 15 min of incubation ALA is being incorporated by diffusion, and after that time transport is mediated by an active system.

Figure 4

**Figure 4:**
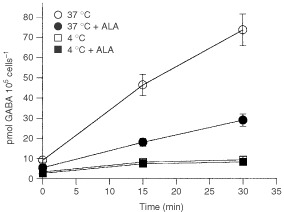
Dependence of GABA uptake on temperature. GABA uptake was measured at 37°C or 4°C in the absence or the presence of ALA. When uptake was determined in the presence of ALA, cells were preincubated 15 min with 10 mM ALA. Then ^14^C-GABA was added and radioactivity was measured.

shows that the initial uptake rate of GABA at 4°C is not significant and it is not altered by the presence of ALA, indicating that GABA diffusion is negligible.

## DISCUSSION

We demonstrate here that ALA is incorporated into murine mammary adenocarcinoma cells by two different processes. One of these processes is passive diffusion which is significant at shorter incubation intervals. The other is an active transport system which becomes very important after the first 15 min of incubation. Recently, it was found that incorporation of Me-ALA, a more lipophilic derivative of ALA, is also mediated by at least two different mechanisms, passive diffusion and active transport (Gederaas *et al*, 2001). In contrast, other authors (Rud *et al*, 2000) reported that ALA is only uptaken by an active process while diffusion is negligible. Döring *et al* (1998) have shown that ALA is a substrate for both mammalian intestinal and renal PEPT1 and PEPT2 transporters. In rat pancreatic tumour cells ALA uptake is mediated by PEPT1 (Whitaker *et al*, 2000). Novotny *et al* (2000) also reported that PEPT2 is one of the two transporters responsible for ALA incorporation at the choroid plexus. Results here presented indicate that in our murine mammary adenocarcinoma cell cultures ALA is not incorporated by any of these transporters since their substrates, the dipeptide gly-gly, the tripeptide gly-gly-ala and the angiotensin-converting enzyme inhibitor captopril (Boll *et al*, 1996) do not significantly block ALA uptake.

Moreover, in our cell system ALA incorporation appears not to be mediated either by the amino acid transport systems A, ASC, GLY, IMINO, PHE and B° because the substrates for these transporters, that is methyl-aminoisobutyrate (alfa-Me-AIB), alanine, glycine, proline and phenylalanine (Palacín *et al*, 1998) do not significantly affect ALA incorporation.

ALA methyl ester does not inhibit ALA uptake, in agreement with Rud *et al* (2000) and Gederaas *et al* (2001), who reported that ALA and its derivative ALA methyl ester, do not share the same transport system.

We found that ALA uptake is inhibited to the highest extent by GABA and β-amino acids, β-alanine and taurine, suggesting that the BETA transporters are involved in ALA transport. BETA transporters are GAT-1 to GAT-3, BGT-1 and TAUT (Palacín *et al*, 1998). We have observed that betaine, the substrate of BGT-1, has no effect on ALA uptake and neither has methyl-AIB, an inhibitor of TAUT system (Palacín *et al*, 1998). Consequently, GAT-1 to GAT-3, the high-affinity GABA transporters, seem to be the best candidates for ALA transport in our murine mammary adenocarcinoma cell system.

The strong inhibition of ALA transport by GABA and viceversa support this proposal. Using human colon adenocarcinoma cells, Rud *et al* (2000) have already shown that ALA is incorporated by BETA transporters. We should also recall that in the yeast *Saccharomyces cerevisiae* ALA and GABA share the UGA4 transport system (Bermúdez Moretti *et al*, 1996).

Among the high-affinity GABA transporters, GAT-2 is the only one expressed in peripheral tissues in addition to brain and retina (Borden *et al*, 1992; Liu *et al*, 1993; Ikegaki *et al*, 1994), therefore it is very likely that GAT-2 is also the ALA transporter in murine mammary adenocarcinoma cells. However, both the regulation of gene expression and substrate specificity in neoplastic cells may be different from the corresponding normal tissue; if so, these differences may be exploited to enhance ALA-PDT selectivity.

Despite the great similarities between ALA and GABA transport systems, the lack of diffusion of GABA in our cell system represents the main distinct feature.

The elucidation of ALA transport mechanisms in tumoural cells would be of major scientific interest and importance for the design of new ALA derivatives which can be expected to more easily penetrate into these cells. The relationship between ALA and GABA transport systems would help to develop new prodrugs, by taking into account the structures of GABA transport competitors (Krogssaard-Larsen *et al*, 2000; Soudijn and van Wijngaarden, 2000). This approach could be of particular interest in the photodynamic treatment of tissues with high GAT-1 to GAT-3 expression such as glioblastomas and neuroblastomas.
